# Massachusetts Veterinary Association

**Published:** 1898-02

**Authors:** Henry S. Lewis

**Affiliations:** Secretary


### MASSACHUSETTS VETERINARY ASSOCIATION.

THE regular monthly meeting was held November 24, 1897, at 19 Boylston Place, Boston, President Winchester in the chair. Members present: Drs. Beckett, Burr, Cronon, Frothingham, Hamilton, Labaw, Lee, Lewis, Paige, McLaughlin, Parker, Pierce, Rogers, Sheldon, Soule, Stickney, Winchester, and Winslow.

Dr. J. B. Paige, of the Massachusetts Agricultural College, Amherst, Mass., read a paper on "Veterinary Education in European Schools" that he visited.

The Association had as guests Mr. Paige, a brother of the lecturer, and Drs. J. S. Cutting, of Medford, and Charles H. Higgins, of Dover.

HENRY S. LEWIS,
Secretary.

